# An intracardiac accessory thyroid gland mimicking cardiac tumor: A case report and literature review

**DOI:** 10.1097/MD.0000000000009465

**Published:** 2017-12-22

**Authors:** Lin-Jing Wang, Chun-Mei Liu, Xin Chen, Li Zhang, Hong-Wei Zhou

**Affiliations:** a Department of Radiology, The First Hospital of Jilin University; b Department of Gynecology, Obstetrics and Gynecology Hospital of Changchun City, Changchun, China.

**Keywords:** cardiac tumor, intracardiac accessory thyroid

## Abstract

**Rationale:**

An accessory thyroid gland (ATG) in the right ventricle is an extremely rare condition. Described herein are histological findings of ATG in the right ventricle found in a patient with a normal cervical thyroid gland.

**Patient concerns:**

A 53-year-old woman was referred to our hospital after experiencing intermittent precordial pain for 2 years.

**Diagnoses:**

The mass in the right ventricle was diagnosed pathologically as ATG.

**Interventions:**

Complete excision was performed because of the patient's intermittent precordial pain and to exclude the possibility of malignancy.

**Outcome:**

The patient's pain was resolved. No recurrence was observed during the 6-month follow-up.

**Lessons:**

After review and analysis of the case, we found that plain and contrast-enhanced computed tomography scans showed that the mass had a similar intensity and enhancement to a cervical thyroid gland, which we think may be a useful clue for making a preoperative diagnosis of ATG.

## 1 Introduction

An ectopic thyroid gland (ETG) has a prevalence of approximately 1 per 100,000 to 300,000 persons, and it reportedly occurs in 1 in 4000 to 8000 patients with thyroid disease.^[1]^ The male-to-female ratios are l:3 to 1:8.^[2]^ Seventy percent of patients with displaced thyroid tissue lack a cervical thyroid gland.^[3]^ More than 90% of reported ETGs are lingual thyroids occurring at the base of the tongue.^[4]^ Thus, a benign intracardiac accessory thyroid gland (ATG) with a cervical thyroid gland is a rare but fascinating congenital deformity. Despite its rarity, ATG must be considered in the differential diagnosis of an intracardiac tumor.

## 2 Case presentation

**Figure 1:**
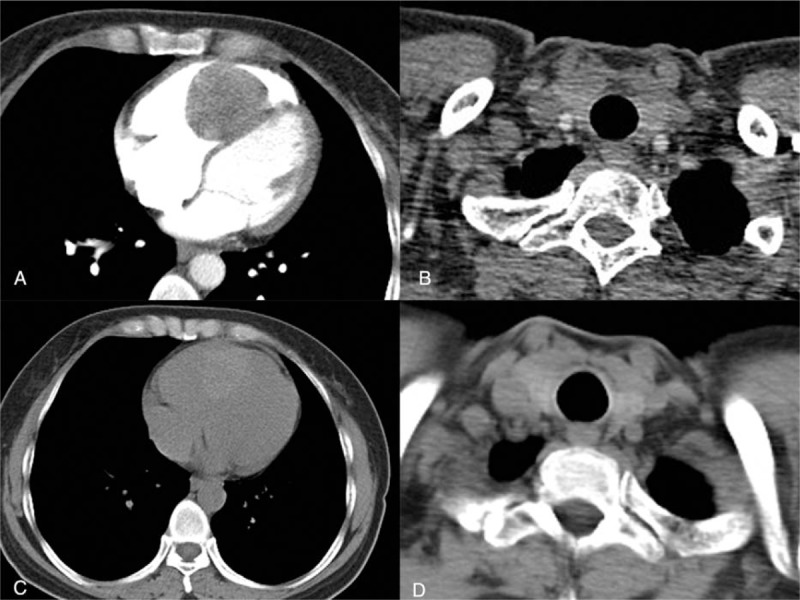
Imaging characteristics of the ectopic thyroid gland in our patient. (A, B) A filling defect is noted in the right ventricle on a contrast-enhanced computed tomography (CT) scan. The mass has a similar enhancement as the cervical thyroid. (C, D) The mass has a similar intensity to the cervical thyroid on the plain CT scan. CT = computed tomography.

**Figure 2:**
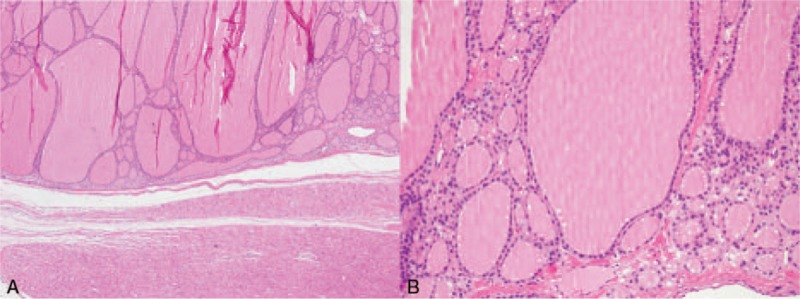
(A) Some myocardial tissue is the accessory thyroid. There are no malignant features and no evidence of endomyocardial invasion (magnification ×40). (B) The mass in the right ventricle has well-differentiated thyroid follicular tissue (magnification ×200).

A 53-year-old woman was referred to our hospital after experiencing intermittent precordial pain for 2 years. No related treatment was given, and she denied relevant heredopathia. The physical examination showed a blood pressure of 121/79 mm Hg (16.1/10.5 kPa) and a heart rate of 80 beats per minute. The computed tomography (CT) scan revealed a tissue measuring 4.8 × 4.1 cm that appeared to be attached to the ventricular septum and to have infiltrated the wall of the right ventricle. A contrast-enhanced CT scan of the chest revealed a filling defect in the right ventricle, which was considered a probable cardiac tumor of the right ventricle (Fig. 1A–D). The laboratory test and imaging study did not show remarkable findings, so to exclude malignancy and other masses, the tumor was surgically removed. Thoracotomy showed a significantly expanded right ventricle as well as a mass broadly attached to the middle valvula tricuspidalis with a smooth mahogany surface, elastic consistency, and no invasion. Surprisingly, the histopathological study demonstrated well-differentiated thyroid follicular tissue in the right ventricle, with an intact capsule and extracapsular myocardium. There were no malignant features and no evidence of endomyocardial invasion (Fig. 2A and B). A diagnosis of ETG was made based on these characteristic features. However, the patient had a normal cervical thyroid gland, so we also diagnosed her as having an ATG.

After surgery, results of a thyroid function test showed subclinical hypothyroidism. Endocrine therapy was conducted for 3 months. The patient is currently free of disease and complaints.

## 3 Discussion

The pathogenesis of an ETG remains unknown in most cases. ETGs are rare entities that result from developmental defects in the early stages of thyroid gland embryogenesis and can occur in any part of the body.^[4]^ ETGs away from the midline are rare, not to mention in the heart, such as in our patient. Although intracardiac ETGs have been described previously and reviewed, to our knowledge, this is the first description of a benign cardiac ETG imaged with both plain CT and contrast-enhanced CT.

ETG can occur in a variety of possible locations, and its clinical appearance differs according to its location and size. The variable symptoms render ETG difficult to diagnose. In our case, the patient had experienced intermittent precordial pain because of a mass in the right ventricle that affected blood circulation. We originally suspected the mass to be a cardiogenic tumor. It was not at first deemed meaningful that the mass in the right ventricle had a similar intensity to the cervical thyroid gland on both the plain and contrast-enhanced CT scans, which led to a misdiagnosis preoperatively.

Whether a patient with an ETG requires treatment and which treatment method (e.g., medication or excision) should be used depend on the nature, size, symptoms, thyroid function, and most important, if the normal cervical thyroid gland, is present, because the ETG could be the only functioning thyroid tissue. Fortunately, in our case, the surgical removal of the ATG had much less of an effect on the patient's thyroid function during the presentation of a cervical thyroid gland. This case highlights the need for a definite preoperative diagnosis of a normal thyroid position to enable clinicians to accurately assess the risks and potential benefits of ETG resection.

Even it is exceptional, an ATG should also be considered when making the diagnosis of intracardiac masses or masses of other parts of the body, especially when the mass has a similar intensity to the cervical thyroid gland on both plain and contrast-enhanced CT scans. Our case provides a meaningful clue to diagnosing ATG. A series of more comprehensive medical examinations, such magnetic resonance imaging, and I131 thyroid scintigraphy, are valuable in providing high sensitivity and specificity for diagnosing ATG.
